# Effects of Extended Treatment with *Protium heptaphyllum* Liposomes on Metabolic Parameters of Obese Rats

**DOI:** 10.3390/biology13100771

**Published:** 2024-09-27

**Authors:** Naiéle Sartori Patias, Sara Vieira Maia, Yasmin Gabriele Ferreira, Natalhya Letícia Ferreira de Oliveira, Stela Regina Ferrarini, Gisele Facholi Bomfim, Adilson Paulo Sinhorin, Danilo Henrique Aguiar, Eveline Aparecida Isquierdo Fonseca de Queiroz, Valéria Dornelles Gindri Sinhorin

**Affiliations:** 1 Programa de Pós-Graduação em Biotecnologia e Biodiversidade (Rede Pró-Centro-Oeste), Universidade Federal de Mato Grosso, Sinop 78550-728, MT, Brazil; nai.sartori@gmail.com (N.S.P.); adilson.sinhorin@ufmt.br (A.P.S.); 2 Instituto de Ciências da Saúde, Universidade Federal de Mato Grosso, Sinop 78550-728, MT, Brazil; saravieiramaia@gmail.com (S.V.M.); yasminferreiray@gmail.com (Y.G.F.); natalhyaleticia56@hotmail.com (N.L.F.d.O.); stela.ferrarini@ufmt.br (S.R.F.); gisele.bomfim@ufmt.br (G.F.B.); 3 Programa de Pós-Graduação em Ciências em Saúde, Universidade Federal de Mato Grosso, Sinop 78550-728, MT, Brazil; 4 Instituto de Ciências Naturais, Humanas e Sociais, Universidade Federal de Mato Grosso, Sinop 78550-728, MT, Brazil; dha.danilo@gmail.com; 5 Departamento de Química, Instituto de Química, Universidade Federal de Mato Grosso, Cuiabá 78060-900, MT, Brazil

**Keywords:** quercetrin, phytotherapeutic treatment, oxidative stress, hypercaloric diet

## Abstract

**Simple Summary:**

*Protium heptaphyllum* (*P. heptaphyllum*), a plant popularly known as “almacega” or “white pitch”, has been used for decades in folk medicine, mainly due to its anti-inflammatory and analgesic properties, and is already recognized due to the use of its resin. Given that obesity is considered a worldwide epidemic, associated with chronic low-grade inflammation and increased reactive oxygen species, this study investigated the effects of *P. heptaphyllum* leaves due to their richness in flavonoids and antioxidant compounds. Using a model of induced obesity in rats, the animals were treated for 28 days with liposomes containing the extract of *P. heptaphyllum* leaves. The results indicate that the treatment may have therapeutic potential not only against obesity, but also in the regulation of oxidative stress and metabolic and inflammatory parameters, possibly due to the high concentration of flavonoids present in the extract.

**Abstract:**

*Protium heptaphyllum* (*P. heptaphyllum*), popularly known as “almacega” or “white pitch”, is widely used in folk medicine due to its antioxidant, anti-inflammatory and healing properties, attributed to its richness in flavonoids and terpenes. Therefore, this study aimed to evaluate the effects of treatment for 28 days with liposomes containing *P. heptaphyllum* leaf extract in obese animals. Male Wistar rats, subjected to a hypercaloric diet for 8 weeks to induce obesity (hypercaloric chow and water enriched with 30% sucrose, ad libitum), were treated with the plant formulation (1 mg kg^−1^day^−1^, via gavage) for 28 days. The study investigated morphological, metabolic, redox state, immunological and histological parameters in adipose and liver tissue. Rats were divided into four groups: control (C), liposomes with extract (H), obese (O) and obese treated with liposomes containing extract (OH). The results indicated that the obese group (O) presented weight gain, hepatic steatosis and alterations in metabolic and inflammatory parameters. However, treatment with liposomes (OH) reduced glucose, alanine aminotransferase (ALT), aspartate aminotransferase (AST), alkaline phosphatase (ALP), creatinine and the lipid profile. In adipose tissue, the OH group showed decreased superoxide dismutase (SOD) activity and increased glutathione S-transferase (GST) activity, in contrast to the effects observed in liver GST. In the analysis of thiobarbituric-acid-reactive substances (TBARS), it was possible to observe an increase in all groups in adipose tissue and in group O in liver tissue, in addition to a reduction in TBARS in group OH in the liver, indicating modulation of oxidative stress. The treatment also increased the concentration of IL-10 and IL-17 in the liver and decreased that of IL-6 in adipose tissue. After 28 days of treatment, these results point to the therapeutic potential of treatment with *P. heptaphyllum*, not necessarily only against obesity, but also an effect per se of the liposomes, possibly due to the high concentration of flavonoids present in the plant extract.

## 1. Introduction

*Protium heptaphyllum* (*P. heptaphyllum*) is a plant widely found in South America, especially in the Amazon region, and is popularly known as “almacega” or “white pitch”. Belonging to the Burseraceae family and the *Protium* genus, this plant stands out not only for the quality of its wood, but also for the various applications already described in traditional medicine. Recognized for its stimulating, anti-inflammatory and healing properties, it is frequently used in folk medicine. The natural substances present in *Protium heptaphyllum* increase its potential as a promising source of alternative treatments and natural derivatives, promoting benefits to human health [1,2].

The resins of the *Protium* genus are rich in flavonoids and terpenes, with emphasis on pentacyclic triterpenes. Analysis of the leaves, flowers, bark resin, stem and branches of *P. heptaphyllum* revealed that the composition found in its plant resin is rich in active substances [3]. The essential oils derived from these resins, in turn, are predominantly composed of monoterpenes [2]. The leaves are rich in flavonoids such as myricetin, quercetin and quercetin-3-β-D-glucoside [4]. The chemical diversity found in plants guides researchers to investigate the biological activity of natural products, given the capacity of these plants to serve as promising sources of active principles for the treatment of several chronic and degenerative diseases. These diseases include cardiovascular, neurological and respiratory diseases, rheumatoid arthritis, kidney diseases, and cancer, often associated with oxidative stress [5,6,7]. This stress is the result of increased production of free radicals and decreased antioxidant defense. Free radicals, also known as reactive oxygen species (ROS), are highly reactive molecules with one or more unpaired electrons at the center of oxygen atoms, and are produced in the cytoplasm, mitochondria or plasma membrane [8].

Another health problem that has affected millions of people is related to the dynamics of the modern world and changes in eating behavior, which stand outs as an interaction between the physical state of the organism and environmental conditions [9,10,11]. These changes are linked to the relationship between stress and obesity. Chronic stress is correlated with metabolic disorders and adjustments in energy homeostasis [12], and can trigger pleasurable and compulsive eating behaviors, such as a preference for sweet and fatty foods, thus contributing to the development of several diseases, such as obesity, diabetes and dyslipidemia. An increased prevalence of obesity is intrinsically associated with changes in eating patterns, sedentary lifestyles and obesogenic environments [13]. Furthermore, it is important to know that a complex interplay between genetic and external factors can act in the individual and contribute to the accumulation of adipose tissue and the development of obesity. A deeper understanding of the molecular, metabolic and endocrine mechanisms underlying obesity is essential for the development of strategies to prevent, control and/or treat this pathology. The expansion of adipose tissue contributes to the exacerbated production of adipokines and pro-inflammatory cytokines, triggering an inflammatory state that is closely linked to the development of metabolic comorbidities, such as insulin resistance, type 2 diabetes, dyslipidemia and cardiovascular diseases [14,15,16].

Plants and their extracts have significant importance in the treatment of various diseases, including obesity. Research suggests that the flavonoids found in these extracts can alleviate cellular stress, including neuroinflammation, oxidative stress, proteotoxicity and endoplasmic reticulum stress [17]. Furthermore, several studies demonstrate that flavonoids have beneficial effects when it comes to obesity, mainly due to their antioxidant and anti-inflammatory properties, mitigating complications related to this pathology [18,19,20]. To further enhance these therapeutic effects of natural compounds, the application of nanosystems has shown to be a promising alternative. Studies have extensively investigated the use of nanosystems in the transport and optimization of the pharmacological action of natural products [21]. These systems involve different structures that vary in chemical composition, shape, charge, solubility and load capacity, among other factors [22], offering an approach that improves the solubility, stability and specific targeting of active substances, overcoming absorption and bioavailability challenges [23]. Among nanosystems, liposomes stand out, which are vesicles composed of one or more concentric phospholipid bilayers that surround an aqueous compartment. They have the capacity to transport both hydrophilic and lipophilic substances, such as drugs, biomolecules and/or diagnostic agents [24]. The incorporation of active substances into liposomes not only allows controlled release, but also minimizes side effects and enhances therapeutic effects. This represents a promising frontier for the optimization of phytotherapeutic treatments in contemporary medicine [23,25]. In addition to improving the solubility of natural compounds, this technology enhances their absorption, allowing for more effective exploration of the medicinal properties of natural ingredients. Thus, nanotechnology, especially with the use of liposomes, opens up new perspectives for the formulation of products that maximize the therapeutic potential of plant substances, contributing to significant advances in the health and well-being industry [26,27].

In view of this, and considering previous studies by our group [28] where the treatment with liposomes containing *P. heptaphyllum* leaf extract in rats under induced obesity was investigated, this study aimed to demonstrate the effects of a longer treatment, also with liposomes containing *P. heptaphyllum* leaf extract, in obese animals, in order to verify whether this route of administration for a longer period could present better effects in this experimental model.

## 2. Material and Methods

### 2.1. Extract Preparation and Liposome Development

The study was conducted at the Laboratórios Integrados de Pesquisa em Ciências Químicas (LIPEQ)—Natural Products Chemistry Laboratory and Biochemistry Laboratory, located at the Universidade Federal de Mato Grosso, Campus Universitário de Sinop. The exsiccata is cataloged under number 625 in the Herbarium Centro-Norte-Mato-Grossense (CNMT), also located at the Sinop Campus. Details on the treatment of *P. heptaphyllum* leaves and the development of the liposome containing the extract can be found in Patias et al. [28].

### 2.2. Experimental

#### 2.2.1. Animals

Male Wistar albino rats were used as experimental subjects in the present study, in compliance with the conditional ethical guidelines of the Animal Use Ethics Committee of the Universidade Federal de Mato Grosso, under approval number 23108.031684/2021-21. The animals in the control group were fed a diet composed of a standard rodent chow (NUVILAB CR-1, Nuvital^®^, Colombo, Paraná, Brazil), while the animals in the experimental group were fed a hypercaloric diet, characterized by a high concentration of lipids, representing 24.5% of the total energy intake, in addition to having access to water containing sucrose (300 g L^−1^). The experimental design and diet formulation followed the methodologies described by Nascimento et al. [29] and Comiran et al. [30]. The hypercaloric diet was prepared in the laboratory and included ingredients such as commercial feed (NUVILAB CR-1), casein, lard, condensed milk and cornstarch biscuits, as well as the addition of vitamins and minerals as detailed in Comiran et al. [30]. The acclimatization period lasted 2 weeks, followed by an 8-week study period. During the experimental period, the animals were kept in polypropylene boxes, with 4–5 rats/box, in an environment with temperature and light–dark cycle control (temperature of 22 ± 2 °C and a 12 h light–dark cycle). The animals had free access to water and food during all experimental protocols. The animals were subdivided into 4 groups: Group C: Negative control. Group H: Control treated with *P. heptaphyllum* extract liposomes for 28 days. Group O: Obese. Group OH: Obese treated with *P. heptaphyllum* extract liposomes for 28 days.

In the groups subjected to treatment with liposomes of the extract (H, OH), a dose of 1.0 mg kg^−1^, established by the Malone test [31], was administered via gavage once a day over 30 days (from the 31st to the 60th day of experiment). The control groups (C and O) were treated with saline solution (NaCl 0.9%). After the last day of the treatment and experimental protocol, the animals were fasted for 8 h to collect the blood samples and tissues. Blood collection was performed by cardiac puncture using heparinized syringes under anesthesia (Ketamine 50 mg kg^−1^ and Xilazin 2 mg kg^−1^); then, the animals were euthanized by decapitation. Several tissues were collected by dissection, such as liver and adipose tissue, weighed to determine the absolute tissue weight (g) and relative tissue weight (g 100 g^−1^ of body weight), and frozen at −80 °C for subsequent analyses. In addition, body weight (g) was recorded to evaluate the final body weight of the animals after the treatment protocol. Plasma was obtained from the collected whole blood.

#### 2.2.2. Characterization of Obesity, Calorie, Food and Water Consumption

The relative weight of periepididymal and retroperitoneal fat, in g/100 g of body weight, was assessed at the end of the experiment, as well as the daily consumption of water and food. The protocol to analyze the consumption of food, water and calories was the same as described in [28].

#### 2.2.3. Intraperitoneal Insulin Tolerance Test (IPITT) and Oral Glucose Tolerance Test (OGTT)

The IPITT and OGTT were developed as described in [30]. The animals were evaluated at the end of the experimental protocol, after 28 days of treatment.

#### 2.2.4. Metabolic Parameters in Liver, Adipose Tissue and Plasma

Aspartate aminotransferase (AST), alanine aminotransaminase (ALT), alkaline phosphatase (ALP), amylase, lipase, creatinine, glucose, lactate, total proteins, as well as triglycerides, total cholesterol, and LDL-, HDL- and VLDL-cholesterol of blood plasma were analyzed as described in [28].

For adipose and liver tissues, glucose was determined according to Dubois et al. [32], glycogen according to Bidinotto et al. [33] and lactate was estimated according to Harrower and Brown [34]. Protein content was determined by the method described by Bradford [35], amino acid levels were determined according to Spies [36] and ammonia was measured according to the method of Gentzkow and Masen [37].

Superoxide dismutase (SOD) activity was measured according to Misra and Fridovich [38]. For catalase (CAT) activity, the decomposition of H_2_O_2_ was observed following Nelson and Kiesow [39]. Glutathione S-transferase (GST) activity was measured by the production of glutathione S-2,4-dinitrophenyl [40]. The dosage of the enzyme glutathione peroxidase (GPx) was performed according to the method described by Paglia and Valentine [41]. For reduced glutathione (GSH), the formation of anilide thiolate was evaluated and compared to a standard GSH curve [42], and ascorbic acid (ASA) was measured and compared to an ascorbic acid curve according to Roe [43]. The indirect markers of oxidative damage studied included thiobarbituric-acid-reactive substances (TBARS), which were quantified and compared to an increasing MDA curve [44], in addition to carbonyl proteins (Carbonyl), according to the method of Colombo et al. [45]. The protein content in the dosages, with the exception of ascorbic acid, was determined by the Bradford method [35], using bovine serum albumin as a standard for constructing the calibration curve. The absorbance readings of the samples were performed at 595 nm.

#### 2.2.5. Histological Analyses of Liver Tissue

Liver tissue samples were fixed in buffered formalin, followed by dehydration in different concentrations of ethanol. Subsequently, the samples were embedded in resin and then hardened in Histotech plastic molds. This material was then sectioned into 3 µm cross sections (Leica Microsystems 2245 semi-automatic rotary microtome, Wetzlar, Germany) and stained with hematoxylin and eosin (H&E). The slides were then observed under a trinocular optical microscope (Motic brand, Dongguan, China) with the aid of Motic Images Plus 2.0 software, which was used to capture and analyze the images. The images of the liver were evaluated for the presence of apoptosis and/or necrosis, hepatic steatosis (micro- and macro-vesicular), tissue inflammation with the presence of leukocytes infiltration, fibrosis and hepatocyte degeneration.

#### 2.2.6. Immunological Evaluation

To perform immunological analyses of cytokines, such as tumor necrosis factor alpha (TNF-α), interferon gamma (INF-γ), interleukin 6 (IL-6), interleukin 10 (IL-10), interleukin 17 (IL-17) and interleukin 1β (IL-1β), the enzyme-linked immunosorbent assay (ELISA) methodology was used, using commercial kits from the DIY Elisa^®^ brand, according to the instructions provided by the manufacturer (Labtest^®^, Diagnóstico S.A., Minas Gerais, Brazil).

### 2.3. Data Analysis

The data were initially subjected to the Kolmogorov–Smirnov normality test. Then, a two-way analysis of variance (ANOVA) was realized, and the Tukey test to assess means when more than two groups were present was conducted. When necessary, the Kruskal–Wallis test was used, and Dunn’s test. Statistical significance was set at *p* < 0.05. Data were presented as mean +/− standard deviation or median and total range, depending on the analysis. Statistical analysis was performed using the Graph Pad Prism 8.0 statistical software.

## 3. Results

### 3.1. Anthropometric Measurements and Food Intake

Among the results obtained, we observed an increase in the final weight and weight gain of the animals in groups O (obese) and OH (obese treated with liposomes) compared to group C (control). The consumption of feed and water by the animals differed significantly between the control groups (C and H) and obese groups (O and OH) throughout the experimental period, with feed consumption being lower in the obese groups in relation to the control groups and water consumption being higher in the obese groups when compared to the control groups, and with significantly lower water consumption in the OH group compared to the O group, demonstrating that the liposomes of *P. heptaphyllum* reduced the consumption of water with sucrose in this group. When we analyzed the calorie calculation (kcal day^−1^rat^−1^), consumption was significantly higher in groups O and OH when compared to group C (Table 1).

As for tissue weight, we observed an increase in the weight of the periepididymal and retroperitoneal adipose tissue of animals in groups O and OH compared to group C, but obese animals treated with liposomes had a reduction in retroperitoneal tissue in relation to obese animals. The liver was shown to be enlarged in the obese group compared to the negative control, and the OH group significantly reduced the size of this organ in relation to group O, keeping it similar in size to the control (Table 1).

**Figure 1:**
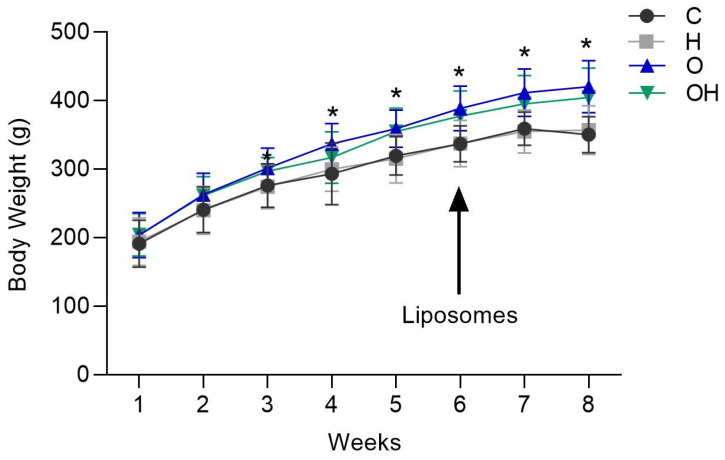
Weight gain of rats in the negative control (C), liposomes (H), obese (O) and obese + liposomes (OH) groups between the 1st and 8th week of the experimental design. Data are expressed as mean ± standard deviation. Analysis was performed by ANOVA (Two-way) and Tukey’s test. * *p* < 0.05 compared with C; (*n* = 8).

Through the analysis of weight evolution from the fourth week of obesity induction, it was possible to observe a significant alteration in the body weight of the rats in the obese groups, with weight being significantly higher in the obese groups (O and OH) in relation to negative group (Figure 1), but no difference was observed between the groups in relation to the treatment with liposomes (C vs. H and O vs. OH) (Figure 1).

**Figure 2:**
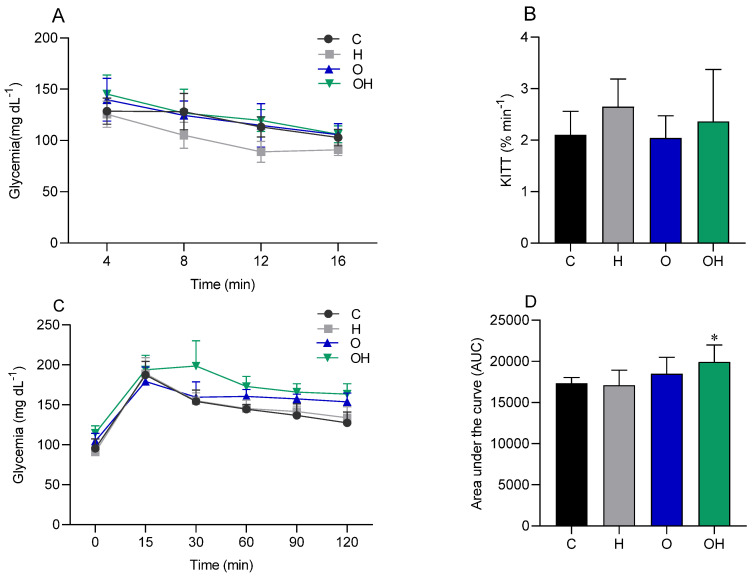
Glycemic curve (**A**) and glucose decay constant (KITT) (**B**), generated by the intraperitoneal insulin tolerance test (IPITT), in the control (C), liposomes (H), obese (O) and obese + liposomes (OH) groups. Glycemic curve (**C**) and area under the curve (**D**), generated by the oral glucose tolerance test (OGTT) of the control (C), liposomes (H), obese (O) and obese + liposomes (OH) groups. Data are expressed as mean ± standard deviation. Analysis was performed by ANOVA (two-way) and Tukey’s test. * *p* < 0.05 compared with C; (*n* = 8).

There was no statistical difference between the groups in relation to the IPITT (Figure 2A,B), demonstrating that there was no difference in insulin sensitivity between the groups. On the other hand, when evaluating the OGTT data, a statistical difference was observed between the OH group and the C group, with results being significantly greater in the OH group (Figure 2C,D).

### 3.2. Analysis of Plasma Parameters

Plasma glucose levels were elevated in groups O and OH in relation to the negative control, and the treatment with liposomes significantly decreased the glucose concentration in the treated obese group compared to the obese group. Total proteins did not change between groups. The activity of the aminotransferases (ALT, AST) and ALP enzymes reduced in the obese groups when compared to the negative control. Creatinine levels were decreased in groups H and OH compared to group C, and the OH group also had a statistically significant reduction when compared to group O. For the dosages of amylase and lipase activities, there was no statistical difference between the groups (Table 2). Regarding the lipid profile data of the animals studied, a decrease in total cholesterol and HDL cholesterol concentrations was seen in the OH group in relation to the negative control. LDL levels were decreased in groups H and OH compared to the C group. VLDL was decreased in H, O and OH compared to group C. We did not observe significant differences in dosages of triglycerides or in the calculation of the triglyceride/HDL cholesterol ratio (TG/HDL) in the groups studied (Table 2).

### 3.3. Analysis of Redox Status Parameters in Adipose and Liver Tissue

**Figure 3:**
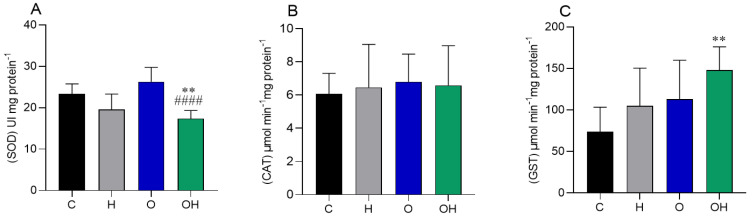
Influence of liposomes containing *P. heptaphyllum* extract on adipose tissue of rats subjected to a hypercaloric diet. SOD: superoxide dismutase (**A**), CAT: catalase (**B**) and GST: glutathione S-transferase (**C**). Data are expressed as mean ± standard deviation. Analysis was performed by ANOVA (two-way) and Tukey’s test. ** *p* < 0.01 compared with C; #### *p* < 0.0001 compared with O; (*n* = 8).

Regarding the oxidative stress biomarkers in adipose tissue, we observed a decrease in SOD activity in the OH group in relation to the negative control and obese groups (Figure 3A). No difference was observed in catalase activity between groups (Figure 3B). The GST enzyme increased its activity in the OH group compared to the C group (Figure 3C).

**Figure 4:**
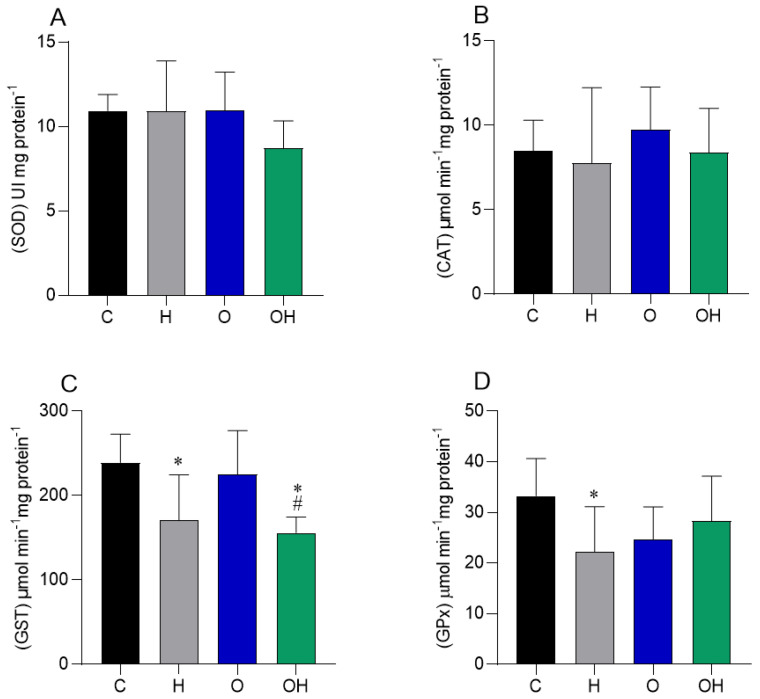
Influence of liposomes containing *P. heptaphyllum* extract on liver tissue of rats fed a hypercaloric diet. SOD: superoxide dismutase (**A**), CAT: catalase (**B**), GST: glutathione S-transferase (**C**) and GPx: glutathione peroxidase (**D**). Data are expressed as mean ± standard deviation. Analysis was performed by ANOVA (two-way) and Tukey’s test. For SOD analysis, the Kruskal–Wallis test was used, and Dunn’s test, with data expressed as median and total range. * *p* < 0.05 compared with C; # *p* < 0.05 compared with O; (*n* = 8).

In the liver tissue, there was a decrease in the activity of the GST enzyme in group H compared to group C, and in group OH compared to groups C and O (Figure 4C). The GPx enzyme decreased its activity in group H compared to group C (Figure 4D). No difference was observed in SOD and CAT activity between groups (Figure 4A,B, respectively).

Regarding markers of lipid damage in adipose tissue, we observed increased levels of TBARS in groups H, O and OH in relation to the negative control. There were no significant changes for the dosages of GSH, ASA and Carbonyl in adipose tissue (Table 3). On the other hand, TBARS levels increased in the liver tissue (O vs. C), but decreased in group OH compared to group O. There was also a decrease in the levels of carbonylated proteins in group O compared to group C in liver tissue. The non-enzymatic markers GSH and ASA did not show alterations in liver tissue (Table 3).

### 3.4. Evaluation of Metabolic Parameters in Adipose and Hepatic Tissue

In the analysis of adipose tissue metabolites, we observed that glucose concentration reduced in the obese rats treated with liposomes compared to the negative control. Glycogen decreased in groups H, O and OH, also compared to group C. For ammonia dosage, we verified an increase in the obese rats and also in the rats treated only with liposomes when compared to the C group, but the treatment reduced this parameter in group OH in relation to group O. Regarding the levels of amino acids, lactate and total proteins, no changes were observed between groups in adipose tissue (Table 4). In addition, we verified an increase in glucose concentration in liver tissue in all groups compared to group C. The concentration of amino acids decreased in groups O and OH in relation to group C. The concentration of ammonia decreased in all treatments in relation to the negative control. Lactate decreased in group O when compared to the C group. For total proteins and glycogen dosages, there were no changes between treatments (Table 4).

### 3.5. Analysis of Cytokines in Adipose and Hepatic Tissues

No changes were observed in the levels of TNF-α and IFN-γ in adipose and liver tissues, as well as no changes in the levels of IL-10, IL-17 and IL-1β in adipose tissue. However, in adipose tissue, IL-6 levels were reduced in groups H, O and OH compared to group C. In liver tissue, an increase in IL-10 was observed in groups H and OH compared to group C, and an increase in IL-17 in groups O and OH compared to group C. No changes were detected in the levels of TNF-α, IFN-γ, IL-6 and IL-1β in liver tissue (Table 5).

### 3.6. Histopathological Evaluation in Liver

**Figure 5:**
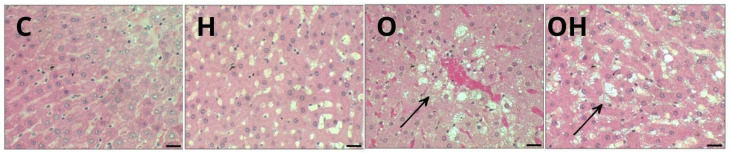
Photomicrograph of a sample of one specimen from each group of liver tissue, stained with hematoxylin and eosin (H&E), showing several morphological alterations in groups C (control), H (Liposomes), O (obese) and OH (Obese + Liposomes). Steatosis: lipid vacuoles in the adipose cytoplasm (arrows) in groups O and OH. Resolution: Bar 30 μm.

Animals fed standard diets (C and H) exhibited a liver with well-formed nucleated hepatocytes, an adequate sinusoidal matrix and an absence of signs such as inflammatory infiltrate, fibrosis or lipid retention, suggesting an architecture compatible with normal liver function (Figure 5, C and H). For all obese rats, it was possible to observe mild steatosis (Figure 5, O and OH).

## 4. Discussion

The therapeutic potential of *P. heptaphyllum* liposomes, rich in flavonoids, was evaluated in this study, which revealed important effects on induced obesity in animals, such as a reduction in liver tissue weight, reductions in glucose, cholesterol, LDL-cholesterol and VLDL-cholesterol in the serum, and a reduction in TBARS levels in the liver. Furthermore, our data presented an increase in final body weight and weight gain of animals in the obese groups when compared to the control group, confirming the success of obesity induction in this model induced by a diet rich in lipids and carbohydrates. Food consumption was reduced in the obese groups throughout the experimental period. However, the consumption of sucrose-rich water was higher in the obese animals, group O, and the administration of liposomes was effective in reducing this consumption in the animals of the OH group. Despite the lower food consumption, the calorie calculation (kcalday^−1^rat^−1^) was higher in the groups that received hypercaloric food, which is consistent with findings from other researchers [28,46,47,48,49].

The results demonstrated that obesity significantly influences the weight of animals, food consumption and distribution of adipose tissue, with an increase in the weight of periepididymal and retroperitoneal adipose tissue in rats subjected to obesity. This corroborates the idea that obesity affects the distribution of adipose tissue, promoting the accumulation of white adipose tissue, mainly visceral, such as retroperitoneal tissue, which is very important in the development of diabetes. Silva et al. [50] and Ribeiro et al. [51], using the same obesity induction model, also observed an increase in body weight and accumulation of visceral white adipose tissue in male Wistar rats. In the present study, prolonged treatment with liposomes did not significantly alter body weight, weight gain or periepididymal adipose tissue compared to the respective control groups (C and O). However, the treatment increased the amount of retroperitoneal adipose tissue in the OH group compared to the obese group. Similar results were observed in studies by Carvalho et al. [52,53], who investigated the effects of α, β-amyrin and *P. heptaphyllum* resin in obese mice induced by a high-fat diet. Both studies, which administered doses of 10 and 20 mg kg^−1^ for 15 weeks, demonstrated a lower increase in body weight, reduced visceral fat accumulation and a significant reduction in net energy intake, suggesting that prolonged treatment with *P. heptaphyllum* liposomes may have beneficial effects in controlling obesity and its metabolic complications, mainly due to the antioxidant and anti-inflammatory properties of the flavonoids present [18,19].

The obese group showed a significant increase in liver weight compared to the control group, along with hepatomegaly, hepatic steatosis (confirmed by histological analysis), increased hepatic glucose and oxidative stress with elevated lipid peroxidation. Treatment with liposomes was effective in significantly reducing liver size compared to the untreated obese group and in decreasing lipid peroxidation, although it did not alter hepatic glucose levels or the steatosis observed histologically. In a previous study using *P. heptaphyllum* liposomes for 14 days [28], such a reduction in liver size and hepatic TBARS levels was not observed, but in the 28-day study, the treatment was more effective, suggesting a time-dependent effect of liposomes in the treatment of obesity and its comorbidities.

No significant differences were observed among groups in relation to the IPITT, which suggests that, even with the increase in body weight in the obese groups, the insulin response may not have been affected at that specific time. When evaluating the OGTT results, the results did not differ between the C vs. H and C vs. O groups, demonstrating that the pancreas of these animals maintains good insulin secretory activity. Furthermore, the absence of statistically significant differences between these groups (C, H and O) during the OGTT indicates that, at that point, no marked differences were observed between them in the body’s ability to metabolize glucose. However, in the OGTT test, it was also observed that treatment with liposomes in the obese group (OH) generated glucose intolerance when compared to the control group. It is possible that the association of obesity with treatment with liposomes generated a synergistic effect leading to glucose intolerance and greater difficulty for the animals in this OH group to secrete insulin when faced with a glucose overload.

Although these results were observed in the IPITT and OGTT, it is important to emphasize that in the fasting state, obese animals presented significant hyperglycemia and that treatment with *P. heptaphyllum* liposomes was effective in correcting this glycemic alteration in OH animals. This suggests a potentially beneficial effect of the extract in reducing plasma glucose in obese animals, and these data are consistent with previous studies with *P. heptaphyllum*, which may be related to a higher level of circulating insulin [4,52,53,54]. Otherwise, in the study by Patias et al. [28] where the treatment of obese animals was for 14 days, glucose levels did not reduce, which leads us to suggest that a longer treatment time is necessary for this hypoglycemic effect to appear (28 days or more). However, as in that study, amylase and lipase, important digestive enzymes, were not modified.

Another parameter that is frequently altered in an obesity condition is the lipidic profile, once the obese frequently presents dyslipidemia. However, we did not observe significant differences between C and O groups in the present work. On the other hand, it was demonstrated that the treatment with liposomes of *P. heptaphyllum* was effective in presenting a hypocholesterolemic effect, reducing significantly the levels of total cholesterol, HDL-cholesterol, LDL-cholesterol and VLDL-cholesterol. The treatment is really significant once it can protect the animals from the development of atherosclerosis. Regarding the triglyceride levels, no difference was observed between groups. These beneficial effects may be related to the presence of flavonoids in *P. heptaphyllum*, such as quercetin-3-β-D-glucoside, myricetin and quercetrin, which are known for their antioxidant properties and other biological effects. Previous studies highlight the role of these bioactive compounds [55,56]. In particular, quercetrin promotes important cardioprotective effects in experimental models exposed to a high-calorie diet [57].

Although triglycerides and the triglycerides/HDL ratio did not change in this experimental model, these findings are interesting because they show that this treatment time resulted in improvements in the lipid profile of the rats in general. When we look at the findings of the 14-day study [28], the results showed some similarities, such as a reduction in LDL and VLDL for groups treated with liposomes. On the other hand, there were no changes in total cholesterol and HDL, but the triglyceride/HDL ratio had an elevation in the obese group treated with liposomes, showing that 14 days was not enough to alleviate these changes. In a study by Santos et al. [54], they emphasized the hypoglycemic effects on the lipid profile obtained by using a mixture of α, β-amyrin extracted from *P. heptaphyllum,* which seemed more prominent at 100 mg/kg, with significant reductions in VLDL and LDL cholesterol and an elevation in HDL cholesterol.

When assessing markers of liver, hepatobiliary and kidney damage, we can observe that all enzymes had their activities reduced in the groups with induced obesity. For creatinine, the groups containing liposomes had a reduction in this marker and it was more reduced in the treated obese group than in the untreated obese group. These findings are in line with the work of Patias et al. [28], as they also observed this reduction in addition to those in aminotransferases and ALP in the obese groups. However, the group treated only with liposomes for AST activity showed an effect per se that did not appear at 28 days. In the current study, on the other hand, at 28 days of treatment, a reduction in creatinine appeared in the treated obese individuals when compared to the untreated obese individuals, which leads us to suggest that with the increase in treatment time, the chance of kidney damage decreases. The increase in aminotransferases and ALP levels is generally associated with liver damage or inflammation, and their decrease may be associated with problems in the production of these enzymes or specific nutritional deficiencies [58,59]. However, as there was induction with a diet poorer in proteins, the decrease is also justified by the lower presence of essential amino acids, necessary for endogenous protein synthesis [60]. Studies carried out by Oliveira et al. [61] showed that *P. heptaphyllum* resin has a hepatoprotective effect in rats intoxicated with acetaminophen, counteracting the increases in ALT and AST, with similar efficacy to N-acetylcysteine (NAC), and also, the investigation done by Patias et al. [4] showed the effects of leaf extract from *P. heptaphyllum* (ethyl acetate fraction) in promoting a reduction in ALT and AST activities in a model of acetaminophen-induced liver injury. Creatinine is the product of muscle phosphocreatine metabolism, and its concentration is related to the use of this substrate by the muscle during vigorous muscle contraction [60], and decreased creatinine levels are also related to other factors that can affect renal and muscle function, such as specific medical conditions, dehydration, medication intake and other individual variations [62].

Adipose tissue is essential in regulating the pathophysiological mechanisms of obesity [63,64]. However, in this study, we did not observe intense changes in enzymatic activities compared to the experimental model examined, but it was possible to observe a reduction in the treated obese group (OH) in SOD and an increase in this same group in GST. In line with this view, the flavonoids present in *P. heptaphyllum* [55], known for their antioxidant and anti-inflammatory properties, can reduce the production of ROS and interfere with enzymatic antioxidant activities [52,53]. The enzymatic changes observed here suggest antioxidant modulation; however, there was an increase in the lipid damage marker (TBARS), suggesting increased oxidative stress in the adipose tissue of these OH animals, and the active ingredients present in the liposomes were not sufficient to neutralize this effect, as observed in 14-day studies with liposomes from the plant [28]. This effect can compromise cellular structures and antioxidant mechanisms, aggravating complications associated with obesity [65]. Studies that used dietary supplementation with antioxidants, such as hydroxy-selenomethionine, showed similar results, where the increase in TBARS was counteracted after the stressor, indicating that different patterns of response to oxidative stress can be modulated depending on the concentration and type of antioxidant present [66].

Mitochondrial dysfunction and chronic inflammation in adipose tissue, exacerbated by oxidative stress, are crucial factors in worsening metabolic complications [67,68,69]. Although no protein damage or changes in non-enzymatic antioxidant parameters (GSH and ASA) were observed, treatment with liposomes for 28 days appears to have prevented the elevation of TBARS levels, unlike what was seen in a previous study with an obese group treated for 14 days [28]. This may be explained by the increase in GST in the OH group, something that was not observed in the 14-day study mentioned above [28], as flavonoids reduce oxidative stress, a key factor in obesity, by increasing antioxidant defenses and reducing inflammation [67].

Obesity may interfere with glucose uptake via the GLUT4 transporter in adipose tissue. In the present study, we observed a reduction in glycogen in the obese groups and also in the H group, in addition to a reduction in glucose levels in the OH group. Although total proteins and amino acids did not show significant variations in the groups studied, we noted a considerable increase in ammonia concentration in the obese and liposome groups, while in the OH group, this concentration was reduced compared to the O group. Considering the possible limited use of glucose as an energy source for the adipocyte, it is possible that part of the energy for this cell comes from the action of oxidative deamination by glutamate dehydrogenase. This process uses glutamate as a substrate and generates α-ketoglutarate, NADH+H^+^ and ammonium ions, the last of which may be releasing its proton into the medium, thus producing ammonia [60]. We do not have findings in the scientific literature that show the effect of flavonoids on ammonia metabolism in adipose tissue, but the study by Patias et al. [28] showed a similar effect, where animals treated with liposomes per se and obese animals showed an increase in ammonia concentration. Notably, when obese animals were treated with liposomes for 14 days, levels of this biomarker were reduced. This result suggests that *P. heptaphyllum* liposomes, possibly due to their ability to improve the bioavailability of active compounds, can influence ammonia metabolism, promoting its reduction in obese states. However, the exact mechanism of interaction with flavonoids still needs to be clarified, although it is already known that they affect the liver, adipose tissue and nervous system, influencing metabolic and signaling pathways related to obesity [70].

Regarding immunological markers, the decrease in IL-6 in treated obese groups indicates a possible downregulation of inflammation, which is in line with previous findings that emphasize that *P. heptaphyllum* resins and leaves exhibit anti-inflammatory activity [28], such as in studies by Siane et al. [71]. Other research has shown that *P. heptaphyllum* extracts can reduce plasmatic concentrations of pro-inflammatory cytokines (TNF-α and IL-6), which are generated by oxidative stress and metabolic dysfunction in obesity, but this effect was not found in this study [52]. Although we did not observe reduced levels of TNF-α, this is consistent with other research that has shown that certain flavonoids, such as those present in blueberry extract, may exert selective anti-inflammatory effects, acting on adipose tissue and specific mediators, such as IL-6, rather than TNF-α. This selectivity may be related to factors such as the dosage or bioavailability of the compounds [72].

The liver is a key organ in the process of metabolizing various exogenous and endogenous substances, and in an obese condition, it becomes vulnerable to changes. When we observed our results, we noticed that GPx and GST had similar reductions in their activities in the H group compared to the control group, and for GST, there was a reduction in the obese group treated with liposomes, which differed from the control and the obese group. Furthermore, the obesity model induced in rats caused an increase in TBARS, indicating increased lipid peroxidation. However, these levels were reduced in the OH group, but with no effect on ASA, SOD and CAT. GST is a very important enzyme in the detoxification of xenobiotics, and like GPx, it is responsible for the detoxification of lipid hydroperoxides (LOOH) [73]. The increase in TBARS seen in obese individuals, reflecting increased oxidative stress and lipid damage, found at reduced levels in the obese group, suggests that treatment with *P. heptaphyllum* liposomes may be able to protect against oxidative stress generated by obesity, as shown in studies linking obesity to an increase in oxidative biomarkers (MDA and LOOH) [74]. GST and GPx activity were also reduced in the H group per se, indicating a possible deficiency in the detoxification of substances and neutralization of hydrogen peroxide as emphasized by Zang et al. [75]. Unlike the study by Patias et al. [28], which found no changes in the activities of hepatic enzymatic antioxidants (SOD, GPx, CAT and GST), non-enzymatic antioxidants (GSH and ASA) and no changes in protein damage (protein carbonylation assay), our 28-day study with liposomes revealed changes in enzymes (GST and GPx), GSH levels and protein carbonylation. This suggests that a longer period of treatment may be necessary to impact liver redox status parameters.

Obesity can also generate increases in glucose in the liver tissue, as a possible route, due to the presence of glycerol from adipose tissue, which can be obtained from the triacylglycerol cycle that produces glucose through hepatic gluconeogenesis [60] but without being transformed into glycogen, as can be observed in the obese groups. In addition, amino acid levels were reduced in the obese groups. In this context, we noticed a decrease in ammonia concentration in the obese and liposome-treated groups, which may reflect a lower activation of the urea cycle. The effects observed per se in group H, namely the increase in glucose dosage as well as the reduction seen in ammonia, suggest that the flavonoids present, mainly quercetin-3-β-glucoside, which contains a sugar residue, may be involved in this increase [55]. As for ammonia, the observed reduction indicates an improvement in the liver’s ability to eliminate toxic compounds, just as the decrease in lactate for the treated obese groups suggests a complex metabolic modulation leading to metabolic stress or adaptation [76]. These findings are consistent with those observed by Patias et al., who also reported similar results in animals treated for 14 days [28]. However, liposomes showed a positive effect in restoring lactate levels in obese individuals, which reinforces the importance of liposome treatment in modulating metabolic parameters altered by obesity.

Considering the already described antioxidant and anti-inflammatory properties of flavonoids present in *P. heptaphyllum*, and their ability to modulate enzymatic antioxidant activities, controlling ROS production, we suggest that the action of the compounds present in the liposomes in this study can reduce oxidative stress and the formation of toxic metabolites, possibly through the suppression of liver cytochrome P450 [52,53,55,77]. Previous studies with *P. heptaphyllum* have demonstrated its therapeutic action in the treatment of liver diseases [4,78,79], corroborating the idea that its bioactive compounds exert hepatoprotective effects. We observed in the liver tissue an increase in IL-17 only in the treated and untreated obese groups, suggesting a chronic inflammatory process activated in this tissue, in this condition. However, we also observed an increase in IL-10, a potent anti-inflammatory cytokine, in the liver of the groups treated with liposomes, which indicates an anti-inflammatory action of this extract even in the absence of obesity. The increase in IL-10 indicates a modulation of the immune system, potentially protecting against chronic liver inflammation while trying to control liver stress [80,81]. These findings highlight the potential of *P. heptaphyllum* in the prevention and treatment of obesity-related and non-obesity liver diseases. Furthermore, the administration of the flavonoids discussed has shown to be a promising strategy to increase the bioavailability of and more efficiently regulate pro-inflammatory cytokines, such as IL-17, offering even greater therapeutic potential in inflammatory diseases [82].

A distinctive feature of this study was the administration of a relatively low dose of 1 mg/kg of the extract, in liposomal form, for a period of 28 days. Even with this reduced dosage, the results were significant, especially when compared to previous studies that used higher doses of flavonoids or amyrins, such as those by Carvalho [52,53] and Patias [4], whose doses ranged from 10 to 20 mg/kg and 100 mg/kg, respectively. The observed efficacy reinforces the potential of *P. heptaphyllum* liposomes, suggesting that this formulation enhances therapeutic effects, offering a promising and innovative approach in the treatment of obesity.

## 5. Final Considerations

Our study revealed that obesity induces significant changes in hepatic metabolism and inflammatory response, as evidenced by increased glucose and ammonia levels, and by changes in the activity of antioxidant enzymes such as GPx and GST. Flavonoids present in *P. heptaphyllum* liposomes appear to positively influence oxidative processes in adipose and hepatic tissues, with decreased SOD activity and increased GST, in addition to variations in lipid damage markers. The decrease in IL-17 and increase in IL-10 in the groups treated with *P. heptaphyllum* suggest a positive modulation of the inflammatory response, potentially protecting the liver from chronic inflammation and helping to control hepatic stress. These findings suggest the need for further investigations to explore in more depth the therapeutic role of this treatment in order to control and treat complications caused by obesity.

## Figures and Tables

**Table 1:** Food ingestion and anthropometric markers.

	Negative Control (C)	Liposomes (H)	Obese (O)	Obese + Liposomes (OH)
Initial body weight (g) (1st day)	163.12 ± 9.85	178.28 ± 35.56	170.12 ± 42.22	174.12 ± 29.48
Body weight (g) (31st day)	194.31 ± 37.63	198.51 ± 34.42	200.62 ± 36.98	197.95 ± 29.89
Final body weight (g) (60th day)	349.48 ± 29.85	355.25 ± 32.09	413.58 ± 38.85 **	406.22 ± 48.57 *
Weight gain (g)	162.21 ± 13.81	148.51 ± 19.08	195.21 ± 12.66 *	201.60 ± 37.12 *
Feed consumption (g day^−1^rat^−1^)	23.33 ± 1.68	24.06 ± 1.59	17.55 ± 1.93 ****	18.06 ± 1.79 ****
Water intake (mL day^−1^rat^−1^)	36.61 ± 2.28	38.99 ± 2.43	55.31 ± 5.11 ****	52.74 ± 7.27 ****^/####^
Calorie intake (kcal day^−1^rat^−1^)	96.04 ± 15.36	94.03 ± 11.64	157.70 ± 22.29 ****	150.60 ± 36.22 ****
Periepididymal adipose tissue (g)	4.42 ± 0.69	5.07 ± 0.60	9.12 ± 1.15 ****	9.08 ± 1.58 ****
Retroperitoneal adipose tissue (g)	6.00 ± 1.22	5.10 ± 0.93	12.01 ± 2.91 ***	15.82 ± 3.39 ****^/#^
Liver (g)	10.77 ± 0.84	10.96 ± 1.19	13.23 ± 0.63 ****	11.58 ± 0.80 ^##^

Results are expressed as mean ± standard deviation. Analysis was performed by ANOVA (Two-way) and Tukey’s test. * *p* < 0.05 compared with C; ** *p* < 0.01 compared with C; *** *p* = 0.0001 compared with C; **** *p* < 0.0001 compared with C; ^#^ *p* < 0.05 compared with O; ^##^ *p* < 0.01 compared with O; ^####^ *p* < 0.0001 compared with O.

**Table 2:** Plasma biochemical parameters.

	Negative Control (C)	Liposomes (H)	Obese (O)	Obese + Liposomes (OH)
Glucose (mg dL^−1^)	117.00; 53.00	121.50; 17.00	188.00; 21.00 ****	154.00 ± 55.00 ****^/##^
Total proteins (mg dL^−1^)	6.56; 7.68	6.55; 2.48	6.14; 3.79	63; 3.54
ALT (U L^−1^)	71.50; 40.00	60.50; 11.00	44.50; 11.00 **	40.00; 19.00 ****
AST (U L^−1^)	188.00 ± 53.37	133.90 ± 27.65	118.10 ± 43.22 *	125.30 ± 41.62 *
ALP (U L^−1^)	176.10 ± 28.29	162.50 ± 18.30	110.10 ± 14.89 ****	111.80 ± 17.89 ****
Creatinine (U L^−1^)	1.30 ± 0.19	0.86 ± 0.12 **	1.06 ± 0.31	0.65 ± 0.16 ****^/##^
Amylase (U L^−1^)	623.80 ± 117.30	644.50 ± 78.89	680.40 ± 61.02	713.18 ± 58.05
Lipase (U L^−1^)	25.00 ± 5.90	19.75 ± 2.65	21.63 ± 6.18	25.88 ± 3.52
Cholesterol (mg dL^−1^)	149.60 ± 17.20	130.00 ± 19.49	135.90 ± 20.67	116.10 ± 16.36 **
HDL (mg dL^−1^)	46.38 ± 6.18	40.75 ± 7.20	40.88 ± 3.27	36.38 ± 3.88 *
LDL (mg dL^−1^)	94.68 ± 20.57	73.46 ± 10.07 *	78.28 ± 11.76	73.10 ± 12.11 *
VLDL (mg dL^−1^)	21.43 ± 6.26	13.78 ± 3.18 **	13.20 ± 3.09 **	15.88 ± 2.44 **
Triglycerides (mg dL^−1^)	74.00; 93.00	72.00; 46.00	69.00; 48.00	53.00; 38.00
TG/HDL (mg dL^−1^)	1.67; 1.93	1.50; 0.67	1.75; 0.44	1.51; 1.14

Data are presented as mean ± standard deviation. Analysis was performed by ANOVA (two-way) and Tukey’s test. For the quantification of glucose, total proteins, ALT, triglycerides and the TG/HDL ratio, the Kruskal–Wallis test was used, and Dunn’s test, with data expressed as median and total range. * *p* < 0.05 compared with C; ** *p* < 0.01 compared with C; **** *p* < 0.0001; ^##^ *p* < 0.01 compared with O; (*n* = 8).

**Table 3:** Redox status parameters analyzed in adipose and hepatic tissues of rats subjected to a hypercaloric diet and treated for 28 days with *P. heptaphyllum* liposomes.

		Negative Control (C)	Liposomes (H)	Obese (O)	Obese + Liposomes (OH)
**Adipose tissue**	GSH (µmol mg protein^−1^)	34.95 ± 10.42	31.42 ± 10.83	32.48 ± 11.60	21.34 ± 6.40
	ASA (μmol g tissue^−1^)	0.37 ± 0.07	0.34 ± 0.08	0.32 ± 0.05	0.32 ± 0.06
	TBARS (nmol mg protein^−1^)	0.64; 0.48	1.43; 1.56 **	1.38; 0.78 **	1.43; 1.03 **
	Carbonyl (nmol mg protein^−1^)	238.38 ± 90.30	149.77 ± 48.27	170.73 ± 78.59	157.08 ± 56.79
**Liver**	GSH (µmol mg protein^−1^)	19.68 ± 4.20	25.85 ± 6.48	20.26 ± 5.55	15.52 ± 6.83
	ASA (μmol g tissue^−1^)	3.05 ± 0.55	2.89 ± 0.52	2.70 ± 0.27	3.24 ± 0.52
	TBARS (nmol mg protein^−1^)	0.14; 0.05	0.19; 0.13	0.24; 0.04 **	0.10; 0.12 ^####^
	Carbonyl (nmol mg protein^−1^)	15.65; 2.48	13.26; 6.54	12.58; 9.08 *	13.57; 4.63

Data are presented as mean ± standard deviation. Analysis was performed by ANOVA (two-way) and Tukey’s test. For the analysis of TBARS in adipose tissue and liver tissue, as well as carbonyls, the Kruskal–Wallis test was used, and Dunn’s test, with data expressed as median and total range. * *p* < 0.05 compared with C; ** *p* < 0.01 compared with C; ^####^ *p* < 0.0001 compared with O; (*n* = 8).

**Table 4:** Analysis of metabolites in rat tissues (liver and adipose tissue) subjected to obesity and treated with liposomes.

		Negative Control (C)	Liposomes (H)	Obese (O)	Obese + Liposomes (OH)
**Adipose Tissue**	Glucose (μmol g tissue^−1^)	5.28 ± 1.71	2.93 ± 1.87	3.73 ± 2.25	2.69 ± 1.57 *
	Glycogen (μmol g tissue^−1^)	11.44 ± 2.16	6.59 ± 1.85 ***	6.15 ± 1.40 ***	5.52 ± 2.93 ****
	Lactate (μmol g tissue^−1^)	1.59; 1.32	1.25; 1.77	1.74; 1.25	2.24; 0.90
	Total Proteins (mg mL^−1^)	0.55 ± 0.33	0.64 ± 0.32	0.62 ± 0.35	0.61 ± 0.37
	Aminoacids (mmol g tissue^−1^)	0.002 ± 0.001	0.002 ± 0.001	0.002 ± 0.001	0.002 ± 0.001
	Ammonia (μmol g tissue^−1^)	0.38; 0.55	1.28; 0.25 ***	1.22; 0.33 **	0.50; 0.46 ^#^
**Liver**	Glucose (μmol g tissue^−1^)	33.93 ± 10.17	69.96 ± 15.80 **	87.22 ± 22.29 ****	69.59 ± 20.00 **
	Glycogen (μmol g tissue^−1^)	1.58; 0.54	1.89; 1.37	1.68; 0.83	2.06; 1,40
	Lactate (μmol g tissue^−1^)	1.62; 0.87	1.73; 1.66	0.89; 0.85 *	1.29; 0.37
	Total Proteins (mg mL^−1^)	6.67; 0.72	7.08; 7.28	6.84; 4.77	8.14; 8.57
	Aminoacids (mmol g tissue^−1^)	0.108 ± 0.014	0.103 ± 0.015	0.074 ± 0.008 ***	0.061 ± 0.010 ****
	Ammonia (μmol g tissue^−1^)	1.16 ± 0.24	0.74 ± 0.16 **	0.86 ± 0.21 *	0.58 ± 0.24 ****

Data are presented as mean ± standard deviation. Analysis was performed by ANOVA (two-way) and Tukey’s test. For the evaluation of ammonia and lactate in adipose tissue, as well as glycogen, lactate and proteins in liver tissue, the Kruskal–Wallis test was applied, and Dunn’s test, with data expressed as median and total range. * *p* < 0.05 compared with C; ** *p* < 0.01 compared with C; *** *p* < 0.001 compared with C; **** *p* < 0.0001 compared with C; ^#^ *p* < 0.05 compared with O; (*n* = 8).

**Table 5:** Evaluation of cytokines TNF-α, IFN-γ, IL-6, IL-10, IL-17 and IL-1β in adipose and liver tissues.

		Negative Control (C)	Liposomes (H)	Obese (O)	Obese + Liposomes (OH)
**Adipose Tissue**	TNF-α (pg mL^−1^)	3.20 ± 0.33	3.24 ± 0.37	3.10 ± 0.35	3.27 ± 0.25
	IFN-γ (pg mL^−1^)	3.43 ± 0.20	3.39 ± 0.25	3.09 ± 0.35	3.13 ± 0.30
	IL-6 (pg mL^−1^)	3.69 ± 0.13	3.26 ± 0.25 *	3.14 ± 0.29 **	3.22 ± 0.22 **
	IL-10 (pg mL^−1^)	2.65 ± 0.27	2.58 ± 0.27	2.55 ± 0.23	2.68 ± 0.36
	IL-17 (pg mL^−1^)	3.65 ± 0.17	3.58 ± 0.23	3.62 ± 0.16	3.68 ± 0.12
	IL-1β (pg mL^−1^)	2.59 ± 0.25	2.62 ± 0.42	2.61 ± 0.27	2.79 ± 0.23
**Liver**	TNF-α (pg mL^−1^)	4.50 ± 0.05	4.47 ± 0.05	4.57 ± 0.07	4.51 ± 0.04
	INF-γ (pg mL^−1^)	4.32 ± 0.09	4.37 ± 0.11	4.43 ± 0.09	4.46 ± 0.12
	IL-6 (pg mL^−1^)	4.32 ± 0.03	4.36 ± 0.05	4.37 ± 0.06	4.40 ± 0.10
	IL-10 (pg mL^−1^)	2.87 ± 0.31	3.17 ± 0.05 **	3.13 ± 0.03	3.20 ± 0.03 ****
	IL-17 (pg mL^−1^)	5.62 ± 0.04	5.62 ± 0.02	5.66 ± 0.03 *	5.66 ± 0.02 *
	IL-1β (pg mL^−1^)	2.15 ± 0.32	2.44 ± 0.26	2.49 ± 0.38	2.44 ± 0.18

Data are shown as mean ± standard deviation. Analysis was performed by ANOVA (two-way) and Tukey’s test. For the evaluation of IL-6 in adipose tissue and IL-10 and IL-1β in liver tissue, the Kruskal–Wallis test was applied, and Dunn’s test, with data expressed as median and total range. * *p* < 0.05 compared with C; ** *p* < 0.01 compared with C; **** *p* < 0.0001 compared with C. For statistical analysis, data were logarithmically transformed; (*n* = 8).

## Data Availability

The data of this study are available from the corresponding author upon reasonable request.
